# Childhood Obesity and Overweight Are Associated with Higher Risk of Depression and Anxiety: A Cross-Sectional Study in Children Aged 6–9 Years

**DOI:** 10.3390/life15060968

**Published:** 2025-06-18

**Authors:** Konstantinos Papadimitriou, Maria Mentzelou, Sousana K. Papadopoulou, Georgios Antasouras, Georgia-Eirini Deligiannidou, Olga Alexatou, Apostolia Ntovoli, Evmorfia Psara, Vasiliki G. Papadopoulou, Constantinos Giaginis

**Affiliations:** 1 Department of Nutritional Sciences and Dietetics, School of Health Sciences, International Hellenic University, 57001 Thessaloniki, Greece; kpapadimitriou@ihu.gr (K.P.); souzpapa@gmail.com (S.K.P.); deligiannidoueirini@yahoo.gr (G.-E.D.); vasilikipapadopoulou52@gmail.com (V.G.P.); 2 Department of Food Science and Nutrition, School of Environment, University of Aegean, 81100 Myrina, Greece; maria.mentzelou@hotmail.com (M.M.); g.antasouras@gmail.com (G.A.); fnsd23003@fns.aegean.gr (O.A.); fnsd21013@fns.aegean.gr (E.P.); 3 Department of Physical Education and Sport Sciences, Frederick University, 3080 Limassol, Cyprus; aero.na@frederick.ac.cy; 4 Department of Physical Education and Sport Science, Aristotle University of Thessaloniki, 57001 Thessaloniki, Greece; 5 Department of Accounting and Finance, University of Macedonia, 54636 Thessaloniki, Greece

**Keywords:** childhood obesity, maternal obesity, gestational weight gain, exclusive breastfeeding, physical activity, depression, anxiety

## Abstract

**Background/Objectives:** The global prevalence of childhood obesity and overweight is steadily increasing, representing a pressing public health concern due to its persistence during adolescence and adulthood and its association with elevated morbidity and mortality risks. This cross-sectional study was designed to examine the potential association between overweight/obesity and the presence of depressive and anxiety symptoms in children aged 6 to 9 years. **Methods:** A total of 4098 children from various urban and rural regions in Greece were enrolled. Data was collected through maternal questionnaires capturing sociodemographic characteristics, perinatal outcomes, anthropometric measurements, breastfeeding practices, and physical activity levels. Children fulfilled the Children’s Depression Inventory (CDI) and the State-Trait Anxiety Inventory for Children—State form (STAIC-S) to evaluate symptoms of depression and anxiety, respectively. **Results:** Childhood overweight/obesity was independently and significantly associated with a more than two-fold increased likelihood of presenting depressive and anxiety symptoms. Childhood overweight/obesity was also significantly associated with maternal obesity, gestational weight gain, childbirth weight, mode of delivery, exclusive breastfeeding, and children’s physical activity. **Conclusions:** Overweight and obesity in children aged 6–9 years are significantly associated with an elevated risk of psychological distress, including depression and anxiety. These findings underscore the need for targeted public health policies and nutritional interventions aimed at promoting healthy lifestyle practices from early childhood. Educational efforts should also support new mothers in adopting and sustaining health-promoting behaviors to mitigate the long-term consequences of childhood obesity.

## 1. Introduction

Obesity is a growing global health crisis, affecting 1 in 8 people worldwide as of 2022, with adult rates more than doubling and adolescent rates quadrupling since 1990. In that year alone, 2.5 billion adults were affected by overweight, containing 890 million obese individuals, whereas over 390 million children and adolescents at the age of 5–19 were affected by overweight, and 160 million of them were affected by obesity [1,2]. Defined as excessive fat accumulation that influences health, obesity is linked to serious conditions such as diabetes type 2 [3], heart disease [4], Crohn’s disease [5], certain cancers [6], reproductive issues [7], and impaired mobility and sleep [8]. Traditionally diagnosed using body mass index (BMI), recent guidelines now emphasize a more holistic approach, incorporating additional measurements like waist circumference and evidence of organ dysfunction or physical limitations [9,10]. This increase shows the complexity of obesity, especially in children, where classifications now include preclinical stages and are based on growth chart percentiles [11]. The issue is particularly severe in countries like Greece, where over 30% of youth are overweight or obese [12]. The rising prevalence and impact of obesity across age groups underscore the urgent need for early intervention, comprehensive assessment, and global public health action [12].

Depression and anxiety are complex mental health conditions that go beyond occasional sadness or worry, interfering with daily functioning and overall well-being [13]. Depression is marked by persistent sadness, fatigue, irritability, sleep and appetite disturbances, and feelings of worthlessness, while anxiety disorders involve excessive, persistent fear and physical symptoms such as a racing heart and sweating [14]. Though some levels of anxiety are normal and even protective, clinical anxiety is pervasive and often debilitating [13,14]. Both conditions can begin in early childhood and are influenced by a mix of genetic, environmental, and personal factors [15]. Recent global crises, notably the COVID-19 pandemic [16], have sharply increased the prevalence of these disorders, with youth particularly affected [15]. In Greece, studies show that one in three young people report depressive symptoms, and one in seven report anxiety [17]. These insights highlight the urgency of early screening and targeted prevention strategies, as well as the need for longitudinal studies to better understand and address these growing mental health challenges.

Recent evidence underscores a complex, bidirectional relationship between obesity and depression in children and adolescents, with most studies indicating a significant association between them, though causality remains unclear [18]. A systematic review of 27 studies conducted between 2014 and 2021 confirmed that obesity is a consistent risk factor concerning both anxiety and depressive behavior [19]. Based on a Swedish cohort, obese girls experienced 43% greater risks of anxiety and depressive behavior compared to girls in the general population, whereas in boys, the results were similar between cohorts [20]. Notably, the influence of the family environment in this association is insufficiently understood, with only one study addressing this variable [19]. In parallel, a prospective study involving a multidisciplinary lifestyle intervention demonstrated significant improvements in both mental health and cardiometabolic outcomes, particularly in children with obesity [21].

Although there are many factors associated with obesity and depression-anxiety, there is limited data concerning children’s cohorts. Therefore, this cross-sectional study is designed to examine the potential association of child age and gender, maternal pre-pregnancy body mass index (BMI), gestational weight gain, childbirth weight, mode of delivery, exclusive breastfeeding, and child physical activity with overweight/obesity and the presence of depressive and anxiety symptoms in children at the age of 6–9 years.

## 2. Methods

### 2.1. Study Population

The present cross-sectional survey initially involved 4817 children aged 6–9 years and their corresponding mothers, being recruited from ten geographic areas across Greece: Athens, Thessaloniki, Larissa, Patra, Alexandroupolis, Kalamata, Ioannina, Crete, as well as the South and North Aegean islands. Participant assignment was conducted randomly between May 2018 and September 2022, primarily by recruiting mothers from primary education schools. The randomization of recruitment was made by applying to a sequence of random binary numbers (i.e., 001110110 in which 0 signified assignment and 1 not assignment to the survey). Participant recruitments were made by the authors of this research article, who were qualified personnel.

**Figure 1:**
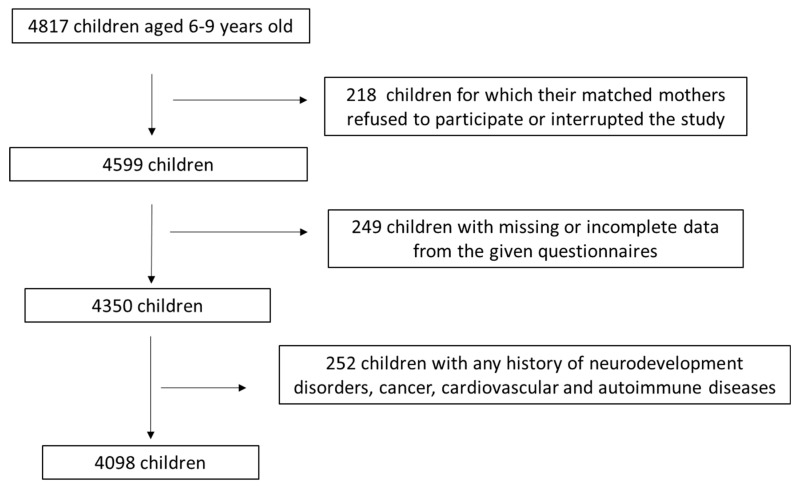
Flow chart diagram of study enrollment.

The initial inclusion criteria required children to be 6–9 years old with no history of chronic disease. Of the 4817 children and their mothers initially recruited, 218 (4.5%) mothers either declined participation or withdrew from the survey before completing the required questionnaires. Among the remaining 4599 participants, 249 (5.4%) were excluded due to missing or incomplete data. Of the 4350 eligible children and their mothers, 252 (5.8%) children were further excluded from analysis due to a reported history of neurodevelopmental disorders, cancer, cardiovascular conditions, or autoimmune diseases. The children’s medical histories were self-reported by their mothers via the survey questionnaires. Consequently, 4098 children and their mothers met all inclusion and exclusion criteria and were included in the last analysis, yielding a final response rate of 85.1%. A flowchart concerning the survey assignment process is depicted in Figure 1.

All children and mothers’ data was severely private. The enrolled mothers were informed regarding the research purposes, signed a consent form, and proved their agreement to the possible publication of their research information namelessly. The survey was approved by the University of the Aegean Ethics Committee (ethics approval protocol: no 12/14.5.2016), being in accordance with the World Health Organization (52nd WMA General Assembly, Edinburgh, Scotland, 2000). The sample size was assessed using PS, the Power and Sample Size calculator program. The determination of the power of the study sample size indicated a power of 87.8%.

### 2.2. Study Design

#### 2.2.1. Sociodemographic Parameters

Throughout the study, relevant questionnaires were utilized for evaluating the sociodemographic characteristics of the enrolled children, such as age, gender (boys vs. girls), nationality (Greek vs. others), and type of residence (urban vs. rural) by face-to-face interviews among their assigned matched mothers and trained personnel to reduce recall biases [22,23]. The maternal education status was categorized into three classes: (a) primary, (b) secondary education, and (c) university studies. The financial level was grouped based on the yearly family income as: 0 EUR < 5000; 1 EUR 5000–10,000; 2 EUR 10,000–15,000; 3 EUR 15,000–20,000; 4 EUR 20,000–25,000; 5 EUR > 25,000. In addition, the economic level was grouped as low for family annual income > EUR 10,000, medium for annual income ˃ EUR 10,000 and ≤EUR 20,000, and high for annual income > EUR 20,000 [22,23]. Mothers’ sociodemographics, such as marital and employment status, smoking habits, and parity, were also collected through face-to-face interviews of the enrolled mothers and the research group to minimize recall biases.

#### 2.2.2. Perinatal Outcomes

Perinatal outcomes, such as gestational weight gain (GWG), childbirth body weight, and the type of delivery (vaginal delivery or Cesarean section), were collected from the mothers’ medical files. According to the Institute of Medicine’s (IOM) guidelines, the suggested GWG for underweight mothers pre-pregnancy (BMI < 18.5 kg/m^2^) ranged from 12.5 to 18.0 kg, for normal-weight women (BMI: 18.5–24.9 kg/m^2^) from 11.6 to 16.0 kg, for overweight women (BMI: 25.0–29.9 kg/m^2^) from 7.0 to 11.5 kg, and for obese mothers (BMI ≥ 30.0 kg/m^2^) from 5 to 9 kg [24]. The enrolled mothers were categorized according to the above criteria into three categories: (a) mothers with a lower than suggested GWG, (b) mothers presenting a normal GWG, and (c) mothers presenting an excessive GWG. Childbirth weight was also taken by mothers’ medical documents, being grouped as low (<2500 g), normal (2500–4000 g), and high (>4000 g) as suggested by the related literature [25]. Mothers’ and children’s weight was measured utilizing a Seca scale [Seca, Hanover, MD, Athens, Greece], without shoes, to the closest 100 g according to the manufacturer’s guidelines, and height was measured utilizing a portable stadiometer (GIMA Stadiometer 27335, Athens, Greece) with no shoes on, to the closest 1 cm according to the manufacture guidelines. The pre-pregnancy mothers’ BMI and anthropometric data throughout the initial weeks of pregnancy were determined by a visit to their gynecologists or public or private hospitals. Both the weight and height data for mothers throughout the first weeks of gestation were extracted from their medical files; thus, they were not self-reported.

#### 2.2.3. Anthropometric Parameters

Children’s anthropometric parameters, such as body weight and height, were measured throughout the survey through trained staff. Body weight was determined by utilizing a similar electronic scale, and height was measured via a portable stadiometer [22,23]. The body weight was determined to the nearest 100 g, and the height was determined to the nearest 0.50 cm. The International Obesity Task Force (IOTF) suggestions were applied to group the allocated children into normal weight, overweight, or obese [26,27].

#### 2.2.4. Breastfeeding Practices

Mothers were asked whether they applied exclusive breastfeeding for at least 4 months. To decrease recall biases, the mothers were responded for exclusive breastfeeding for at least 4 months as they were recommended to steadily contain pulp foods to the feeding strategies of their children at the end of the fourth month and the opening of the 5th month and thus, they can remember more exactly this time point to make their responses more consistent. On the other hand, mothers who breastfed for shorter intervals were not able to respond with sufficient confidence regarding the exact breastfeeding interval [28,29].

#### 2.2.5. Children’s Physical Activity

We assessed children’s physical activity by applying the International Physical Activity Questionnaire (IPAQ), which is short in which participants report the interval of any physical activity during a representative week. This self-administered questionnaire is used worldwide to categorize the total physical activity in the previous seven days as low, moderate, or high [30]. IPAQ instruments have been highly examined and have shown enough reliability and validity, at least as safe as other self-reported PAQs [30]. The Greek version of IPAQ-Short was used in our study, and it was found to present acceptable reliability properties and high repeatability values in young Greek adults [31].

#### 2.2.6. Children Depression and Anxiety

The Children’s Depression Inventory (CDI) was used to determine the presence of depression symptomatology concerning the assigned children [32,33]. This is a self-report assessment written at a first-grade level. The initial 27-item version, which needs from 5 to 15 min for the child to complete, was used. CDI is a well-recognized questionnaire as it is easy to run and score. A child with age-suitable reading abilities can fulfill the scale quite rapidly. CDI exhibits exceptional psychometric properties, as it can evaluate depression in children precisely and reliably when utilized appropriately [32,33]. CDI has an acceptable level of reliability and validity to be implemented in the target community [32,33]. The Greek version of CDI was used in our study, which was found to present acceptable reliability properties and high repeatability values in Greek children [34].

The State-Trait Anxiety Inventory for Children State form (STAIC-S) was used to determine the severity of anxiety symptoms concerning the assigned children [35,36]. STAIC-S was developed by Spielberger and contains 20 items, and it is one of the most utilized self-report tools to evaluate children’s anxiety. The STAIC-S exhibits enhanced consistency and adequate validity [35,36]. The Greek version of STAI-C was used in our study, which has been shown to have acceptable reliability properties and high repeatability values in Greek children [37].

Detailed, comprehensive advice was carefully provided to the mothers and their matched children by trained dietitians and nutritionists concerning the accomplishment of the questionnaires. They also provided a detailed description of the questions to obtain consistent answers and increase the validity of the maternal responses. All questionnaires were completed by the mothers of the assigned children except for the CDI and STAI-S questionnaires.

### 2.3. Statistical Analysis

The continuous variables following normal distribution were analyzed by ANOVA or Student’s *t*-test. The Kolmogorov–Smirnov test was applied to check the normality distribution of the continuous variables. Chi-square was applied for categorical variables. The mean value ± Standard Deviation (SD) was utilized for expressing quantitative variables that follow a normal distribution. The qualitative variables were expressed as absolute or relative frequencies. Multivariate binary logistic regression analysis was applied to evaluate whether children’s overweight/obesity may be independently correlated with sociodemographics, anthropometry, perinatal outcomes, and lifestyle factors, including children’s depression and anxiety, by adjusting for probable confounders. The multiple binary logistic regression results were expressed as odds ratios (OR) and 95% confidence intervals (CI). Differences were recognized as significant at *p* < 0.05 and a 95% CI. The Statistica 10.0 software, Europe, was utilized for the statistical analysis (Informer Technologies, Inc., Hamburg, Germany).

## 3. Results

In Table 1, the descriptive statistics of the survey population are contained. The mean age of children was 7.1 ± 1.1 years old, with a male/female ratio almost equal to 1. Moreover, 95.7% of the children had a Greek nationality, and 66.0% of the children lived in urban areas of Greece. In addition, 30.0% of their mothers had a low educational level, 42.9% had moderate, and 27.1% had a high educational level. Furthermore, 43.1% of children had a low family economic status, 39.8% were moderate, and 17.1% had a high economic status. Additionally, 74.2% of mothers were no smokers, and 69.9% were employed. Lastly, 70.3% of mothers were not divorced, and 63.1% were nulliparous.

Based on pre-pregnancy BMI, 17.2% of the enrolled mothers were overweight, and 4.6% were obese. According to IOM guidelines, 14.7% of mothers were characterized by low gestational weight gain and 38.9% by excessive weight gain. Moreover, 7.9% of children had low birth weight, and 9.4% had high birth weight. In addition, 56.1% of mothers had a cesarean section delivery, while exclusive breastfeeding was applied to 50.1% of children.

Only 12.8% of children had high physical activity, while 39.7% and 47.5% had moderate and low physical activity, respectively. Moreover, 31.8% of children showed depressive symptomatology, and 29.4% of children showed anxiety symptoms.

In cross-tabulation, the potential associations of children’s BMI with the collected variables were examined (Table 2). Overweight/obesity was significantly more frequently observed in girls than boys (*p* ˂ 0.0001). Lower maternal educational level was significantly more often in overweight and obese children (*p* = 0.0269). Family economic status was significantly lowered in children affected by overweight and obesity (*p* = 0.0022). Mothers who were regular smokers had significantly and more frequently overweight and obese children (*p* ˂ 0.0001). Child overweight and obesity were significantly more frequently observed in multiparous (*p* ˂ 0.0001).

Children’s BMI was positively associated with maternal pre-pregnancy BMI status (*p* ˂ 0.0001). Excessive maternal gestational weight gain was more frequently significantly associated with child overweight and obesity (*p* = 0.0002). High childbirth weight was significantly more often in children affected by overweight or obesity (*p* ˂ 0.0001). Children born by Caesarean section were considerably more often overweight or obese (*p* ˂ 0.0001). Children who were not exclusively breastfed were significantly associated with overweight or obesity (*p* ˂ 0.0001).

Low children’s physical activity was significantly associated with overweight and obesity compared to children with moderate or high physical activity (*p* ˂ 0.0001). Overweight and obesity were significantly more frequently observed in children with depressive symptoms (*p* ˂ 0.0001). Overweight and obesity were also significantly more often observed in children with anxiety symptoms (*p* ˂ 0.0001).

In binary logistic regression analysis, child gender, maternal pre-pregnancy BMI status and gestational weight gain, childbirth weight, type of delivery, exclusive breastfeeding, the children’s physical activity, depression and anxiety were significantly and independently associated with the children’s BMI (Table 3, *p* ˂ 0.05). Girls had a 35% higher prevalence of overweight or obesity *p* = 0.0161). Children whose mothers were overweight or obese pre-pregnancy showed an 88% higher risk of being overweight or obese (*p* = 0.0104). Children whose mothers had low or excessive gestational weight gain exhibited a 91% higher probability of being overweight or obese (*p* = 0.0219). Children with low or high birth weight exhibited a 72% greater probability of affecting by overweight or obesity (*p* = 0.0198). Children born by Cesarean section showed a twofold greater risk of being overweight or obese (*p* = 0.0187). Children not receiving exclusive breastfeeding, as well as those with decreased levels of physical activity had a more than two-fold greater probability of being overweight or obese (*p* = 0.0082 and *p* = 0.0061, respectively). Overweight and obese children exhibited a more than twofold greater likelihood of developing depressive and anxiety symptoms (*p* = 0.0072 and *p* = 0.0009, respectively).

## 4. Discussion

This study highlights that among the mothers, 17.2% were overweight, and 4.6% were obese before pregnancy, with 38.9% experiencing excessive gestational weight gain. Cesarean section deliveries occurred in 56.1% of cases, and 50.1% of children were exclusively breastfed. Regarding the children, 7.9% had low birth weight, and 9.4% had high birth weight, while physical activity levels were generally low. Overweight and obesity were more prevalent in girls than boys. Also, overweight conditions were significantly associated with lower maternal education, maternal smoking, multiparity, mothers’ pre-pregnancy overweight/obesity, and unnecessary gestational weight gain. Additional risk factors included cesarean birth, lack of exclusive breastfeeding, low physical activity, and abnormal birth weights. Furthermore, overweight and obese children were more likely to exhibit depressive (31.8%) and anxiety (29.4%) symptoms, indicating a strong association between weight status and mental health.

The present findings highlight a notable gender disparity in childhood overweight and obesity prevalence, with Greek girls exhibiting a 35% higher prevalence than boys. This gender difference aligns with international patterns observed in other populations. For instance, Lindberg et al. [20] reported that girls in a Swedish obese cohort exhibited a 43% greater probability of anxiety and depression in comparison with girls in the general population. In contrast, boys did not show such differences between cohorts. These findings underscore the complex interplay between gender, obesity, and mental health outcomes in children, emphasizing the need for gender-sensitive approaches in addressing childhood obesity.

Maternal factors are critical determinants of childhood overweight and obesity in this study. Consistent with previous research, maternal education, employment, and health behaviors such as smoking were significantly associated with children’s weight status [38]. While the literature presents mixed results regarding maternal education and childhood obesity, often dependent on socioeconomic context, our results contribute to this discussion by revealing a relationship in the Chinese cohort, where higher maternal education correlated with increased BMI Z-scores [39].

Further emphasizing maternal influence, this study found that children born to mothers who smoked regularly exhibited a considerably greater frequency of overweight and obesity in comparison with those whose mothers did not smoke. This aligns with findings by Janjua et al., who reported that maternal smoking of 1–12 cigarettes per day is associated with an increased risk of childhood overweight [40]. Additionally, maternal pre-pregnancy overweight or obesity was linked to an 88% higher risk of childhood overweight or obesity, reinforcing the prevalence of obesity risk [41]. Excessive gestational weight gain also emerged as a significant factor, with children of mothers who gained either insufficient or excessive weight during pregnancy showing a 91% greater likelihood of being overweight or obese, further supporting the critical role of maternal health before and during pregnancy [42].

Birth-related factors also had a significant role in childhood obesity risk. The present study revealed that children born by cesarean section exhibited a 2-fold greater probability of being overweight or obese compared to those born vaginally. Moreover, lack of exclusive breastfeeding was associated with more than double the probability of childhood overweight and obesity, underscoring the protective role of breastfeeding. Mantzorou et al. emphasize the benefits of exclusive breastfeeding for at least four months, not only for reducing childhood obesity risk but also for supporting maternal postpartum weight control, advocating for breastfeeding promotion as a public health priority [22].

Lifestyle behaviors such as physical activity were strongly linked to childhood weight status. Children who did not engage in physical activity exhibited more than twofold overweight or obesity, corroborating findings by Hong et al. that physical activity reduces obesity risk [43]. Furthermore, overweight and obese children had over twice the risk of developing anxiety and depressive symptoms, illustrating the bidirectional relationship between mental health and obesity. These results highlight the need for comprehensive interventions that address both physical and psychological aspects of childhood obesity [44].

A recent systematic review has examined interventional programs that assess the relationship of obesity with depression and their probable influence on the improvement of depression symptomatology during childhood or adolescence, which may affect adulthood [45]. This study has revealed that interventional studies concerning obesity management reveal considerable impacts on depression symptomatology. However, it is speculated that there is heterogeneity in the designs for their standardization and long-term follow-up strategies. Moreover, this study included only children and adolescents aged above 8 years old since there is no data so far for younger children [45]. A meta-analysis by Burke et al. indicated that a small but substantial relationship existed between obesity and anxiety, especially concerning children under 12 years and girls [46]. However, a more recent meta-analysis including more studies (three prospectives and 25 cross-sectional studies) did not find any relation between overweight/obesity and the probability of depression and anxiety in children and adolescents [47]. The above conflicting results reinforce the demand for additional studies on this issue by focusing on more specific age ranges of children and adolescents.

A strength of our survey is the large and representative study population from various areas across Greece, which increases the generalizability of our results. Moreover, we performed careful assessments incorporating diverse sociodemographics, anthropometrics, perinatal outcomes, and lifestyle factors, enhancing the consistency of our research design. To reduce recall bias, we also performed face-to-face interviews with qualified personnel and mothers of the enrolled children, guaranteeing comprehensive explanations and detailed questionnaire descriptions. Anthropometrics were carefully done through trained staff, guaranteeing the precision and consistency of the data over self-reported information.

Nevertheless, there are some limitations to take into consideration when interpreting our results. The cross-sectional design of our survey attenuates our capability to verify causal correlations of childhood overweight/obesity with depression and anxiety. In addition, despite our efforts to reduce recall biases through rigorous face-to-face interviews, possible probabilities of recall biases, specifically concerning the self-reported questions, may exist. For instance, dependence on interviews and medical records may enhance the likelihood of recall biases, especially for variables supported by individual recall, such as breastfeeding strategies. There is also the probability of remaining unmeasured confounding factors, which may affect several aspects of mental health. Hence, it is still probable that residual confounding could affect our findings, although we performed a comprehensive adjustment for several confounding factors.

Overall, this study reinforces the multifactorial nature of childhood overweight and obesity, with significant contributions from gender, maternal and perinatal factors, birth methods, breastfeeding practices, and physical activity. Public health strategies should incorporate tailored, culturally sensitive approaches that address these diverse determinants, focusing particularly on maternal health optimization, breastfeeding promotion, and encouraging physical activity to curb the rising tide of childhood obesity and its associated mental health burdens. School-based interventions designed to prevent anxiety and depression among children and adolescents have been performed, emphasizing that additional research is recommended to gain a more comprehensive understanding of this critical issue [48].

## 5. Conclusions

Overweight and obesity in children aged 6–9 years are significantly associated with an elevated risk of psychological distress, including depression and anxiety. These findings underscore the need for targeted public health policies and nutritional interventions to promote healthy lifestyle practices from early childhood. Educational efforts should also support new mothers in adopting and sustaining health-promoting behaviors to mitigate the long-term consequences of childhood obesity. Moreover, future research should be focused on performing prospective studies to examine whether there is a causal effect of childhood obesity and overweight in the severity of depression and anxiety symptoms.

## Figures and Tables

**Table 1:** Descriptive statistics of the study population.

Characteristics (n = 4098)	Descriptive Statistics
**Age (mean ± SD; years)**	7.5 ± 1.1
**Sex (n, %)**	
Male	2041 (49.8%)
Female	2057 (50.2%)
**Nationality (n, %)**	
Greek	3921 (95.7%)
Other	177 (4.3%)
**Type of residence (n, %)**	
Urban	2703 (66.0%)
Rural	1395 (34.0%)
**Maternal educational level (n, %)**	
Low	1229 (30.0%)
Moderate	1757 (42.9%)
High	1112 (27.1%)
**Family economic status (n, %)**	
Low	1767 (43.1%)
Moderate	1629 (39.8%)
High	702 (17.1%)
**Maternal smoking habits (n, %)**	
No smokers	3041 (74.2%)
Regular smokers	1057 (25.8%)
**Employment status (n, %)**	
Employed	2866 (69.9%)
Unemployed	1232 (30.1%)
**Marital status (n, %)**	
Married	2882 (70.3%)
Divorced	1216 (29.7%)
**Parity (n, %)**	
Nulliparity	2586 (63.1%)
Multiparity	1512 (36.9%)
**Maternal pre-pregnancy BMI status (n, %)**	
Underweight	120 (2.9%)
Normal weight	3085 (75.3%)
Overweight	704 (17.2%)
Obese	189 (4.6%)
**Maternal gestational weight gain (n, %)**	
Low	601 (14.7%)
Normal	1903 (46.4%)
Excessive	1594 (38.9%)
**Childbirth weight (n, %)**	
Low (<2500 g)	324 (7.9%)
Normal (2500–4000 g)	3391 (82.7%)
High (>4000 g)	383 (9.4%)
**Kind of delivery (n, %)**	
Vaginal	1798 (43.9%)
Cesarean section	2300 (56.1%)
**Exclusive breastfeeding (n, %)**	
No	2047 (49.9%)
Yes	2051 (50.1%)
**Children’s physical activity (n, %)**	
Low	1946 (47.5%)
Moderate	1628 (39.7%)
High	524 (12.8%)
**Children’s depression (n, %)**	
No	2253 (68.2%)
Yes	1302 (31.8%)
**Children’s anxiety (n, %)**	
No	2893 (70.6%)
Yes	1205 (29.4%)

**Table 2:** Associations of BMI with sociodemographic and anthropometric parameters, perinatal outcomes, breastfeeding practices, physical activity, depression as well as anxiety.

Characteristics (n = 4098)	BMI			
	Normal 3091 (75.4%)	Overweight 685 (16.7%)	Obesity 322 (7.9%)	*p*-Value
**Age (mean ± SD; years)**	7.53 ± 1.04	7.49 ± 1.08	7.55 ± 1.07	*p* = 0.4428 *
**Sex (n, %)**				*p* ˂ 0.0001 **
Male	1634 (52.9%)	261 (38.1%)	146 (45.3%)	
Female	1457 (47.1%)	424 (61.9%)	176 (54.7%)	
**Nationality (n, %)**				*p* = 0.1673 **
Greek	2968 (96.0%)	649 (94.7%)	304 (94.4%)	
Other	123 (4.0%)	36 (5.3%)	18 (5.6%)	
**Type of residence (n, %)**				*p* = 0.7385 **
Urban	2196 (71.0%)	497 (72.6%)	221 (68.6%)	
Rural	895 (29.0%)	188 (27.4%)	101 (31.4%)	
**Maternal educational level (n, %)**				*p* = 0.0269 **
Low	897 (29.0%)	216 (31.5%)	116 (36.0%)	
Moderate	1328 (43.0%)	291 (42.5%)	138 (42.9%)	
High	866 (28.0%)	178 (26.0%)	68 (21.1%)	
**Family economic status (n, %)**				*p* = 0.0022 **
Low	1313 (42.5%)	300 (43.8%)	154 (47.8%)	
Moderate	1225 (39.6%)	297 (43.4%)	107 (33.2%)	
High	553 (17.9%)	88 (12.8%)	61 (19.0%)	
**Maternal smoking habits (n, %)**				*p* ˂ 0.0001 **
No smokers	2341 (75.7%)	502 (73.3%)	198 (61.5%)	
Regular smokers	750 (24.3%)	183 (26.7%)	124 (38.5%)	
**Employment status (n, %)**				*p* = 0.1148 **
Employed	2186 (70.7%)	468 (68.3%)	212 (65.8%)	
Unemployed	905 (29.3%)	217 (31.7%)	110 (34.2%)	
**Marital status (n, %)**				*p* = 0.8151 **
Married	2166 (70.1%)	486 (70.9%)	230 (71.4%)	
Divorced	925 (29.9%)	199 (29.1%)	92 (28.6%)	
**Parity (n, %)**				*p* ˂ 0.0001 **
Nulliparity	2012 (65.1%)	375 (54.7%)	199 (61.8%)	
Multiparity	1079 (34.9%)	310 (45.3%)	123 (38.2%)	
**Maternal pre-pregnancy BMI status (n, %)**				*p* ˂ 0.0001 **
Underweight	110 (3.6%)	10 (1.5%)	(0.0%)	
Normal weight	2348 (76.0%)	532 (77.7%)	205 (63.7%)	
Overweight	529 (17.0%)	889 (12.8%)	87 (27.0%)	
Obese	104 (3.4%)	55 (8.0%)	30 (9.3%)	
**Maternal gestational weight gain (n, %)**				*p* = 0.0002 **
Low	437 (14.2%)	135 (19.7%)	29 (9.0%)	
Normal	1469 (47.5%)	288 (42.0%)	146 (45.3%)	
Excessive	1185 (38.3%)	262 (38.3%)	147 (45.6%)	
**Childbirth weight (n, %)**				*p* ˂ 0.0001 **
Low birth weight (<2500 g)	269 (8.7%)	49 (7.2%)	6 (1.9%)	
Normal birth weight (2500–4000 g)	2580 (83.5%)	542 (79.1%)	269 (83.5%)	
High birth weight (>4000 g)	242 (7.8%)	94 (13.7%)	47 (14.6%)	
**Kind of delivery (n, %)**				*p* ˂ 0.0001 **
Vaginal	1489 (48.2%)	246 (35.9%)	63 (19.6%)	
Cesarean section	1602 (51.8%)	439 (64.1%)	259 (80.4%)	
**Exclusive breastfeeding (n, %)**				*p* ˂ 0.0001 **
No	1402 (45.4%)	403 (58.8%)	242 (75.2%)	
Yes	1689 (54.6%)	282 (41.2%)	80 (24.8%)	
**Children’s physical activity (n, %)**				*p* ˂ 0.0001 **
Low	1390 (45.0%)	355 (51.8%)	201 (62.4%)	
Moderate	1309 (42.3%)	238 (34.8%)	81 (25.2%)	
High	392 (12.7%)	92 (13.4%)	40 (12.4%)	
**Children’s depression (n, %)**				*p* ˂ 0.0001 **
No	2253 (72.9%)	387 (56.5%)	156 (48.4%)	
Yes	838 (27.1%)	298 (43.5%)	166 (51.6%)	
**Children’s anxiety (n, %)**				*p* ˂ 0.0001 **
No	2230 (72.1%)	466 (68.0%)	197 (61.2%)	
Yes	861 (27.9%)	219 (32.0%)	1259 (38.8%)	

* ANOVA test, ** Chi-square test.

**Table 3:** Multivariate logistic regression analysis for the children’s BMI.

Characteristics	Children BMI (Overweight/Obesity vs. Normal)	
	OR * (95% CI **)	*p*-Value
**Age** (Over/Below mean value)	0.97 (0.23–1.82)	*p* = 0.6131
**Sex** (Male/Female)	1.35 (0.98–1.71)	*p* = 0.0161
**Nationality** (Greek/Other)	1.02 (0.28–1.95)	*p* = 0.7893
**Type of residence** (Rural/Urban)	1.12 (0.34–1.85)	*p* = 0.4381
**Maternal educational level** (Low/Moderate and high)	1.27 (0.71–1.78)	*p* = 0.2873
**Family economic status** (Low/Moderate and high)	1.36 (0.82–1.92)	*p* = 0.1922
**Maternal smoking status** (Regular smokers/No smokers)	1.22 (0.73–1.80)	*p* = 0.1038
**Employment status** (Unemployed/Employed)	1.32 (0.78–1.83)	*p* = 0.2918
**Marital status** (Married/Divorced)	0.98 (0.28–1.93)	*p* = 0.8102
**Parity** (Multiparity/Nulliparity)	1.37 (0.81–1.84)	*p* = 0.0964
**Maternal pre-pregnancy BMI status** (Overweight d obesity/Underweight and normal weight)	1.88 (1.58–2.13)	*p* = 0.0104
**Maternal gestational weight gain** (Low and excessive/Normal)	1.91 (1.42–2.24)	*p* = 0.0219
**Childbirth weight** (Low and high/Normal)	1.72 (1.48–2.09)	*p* = 0.0198
**Kind of delivery** (Caesarean section/Vaginal)	2.12 (1.84–2.39)	*p* = 0.0187
**Exclusive breastfeeding** (No/Yes)	2.41 (2.15–2.68)	*p* = 0.0082
**Children’s physical activity** (Low/Moderate and High)	2.63 (2.31–2.97)	*p* = 0.0061
**Depression** (Yes/No)	2.57 (2.29–2.94)	*p* = 0.0072
**Anxiety** (Yes/No)	2.54 (2.37–2.71)	*p* = 0.0009

* OR—Odds Ratio, ** CI—Confidence Interval.

## Data Availability

The data is available to the corresponding author upon request.
